# Supplementary figures and images for: Circulating Neurofilament Light Chain Levels Increase with Age and Are Associated with Worse Physical Function and Body Composition in Men but Not in Women
Source: Int J Mol Sci. 2023 Aug 13;24(16):12751. doi: 10.3390/ijms241612751 (PMC10454444; doi:10.3390/ijms241612751)

## Slide 1
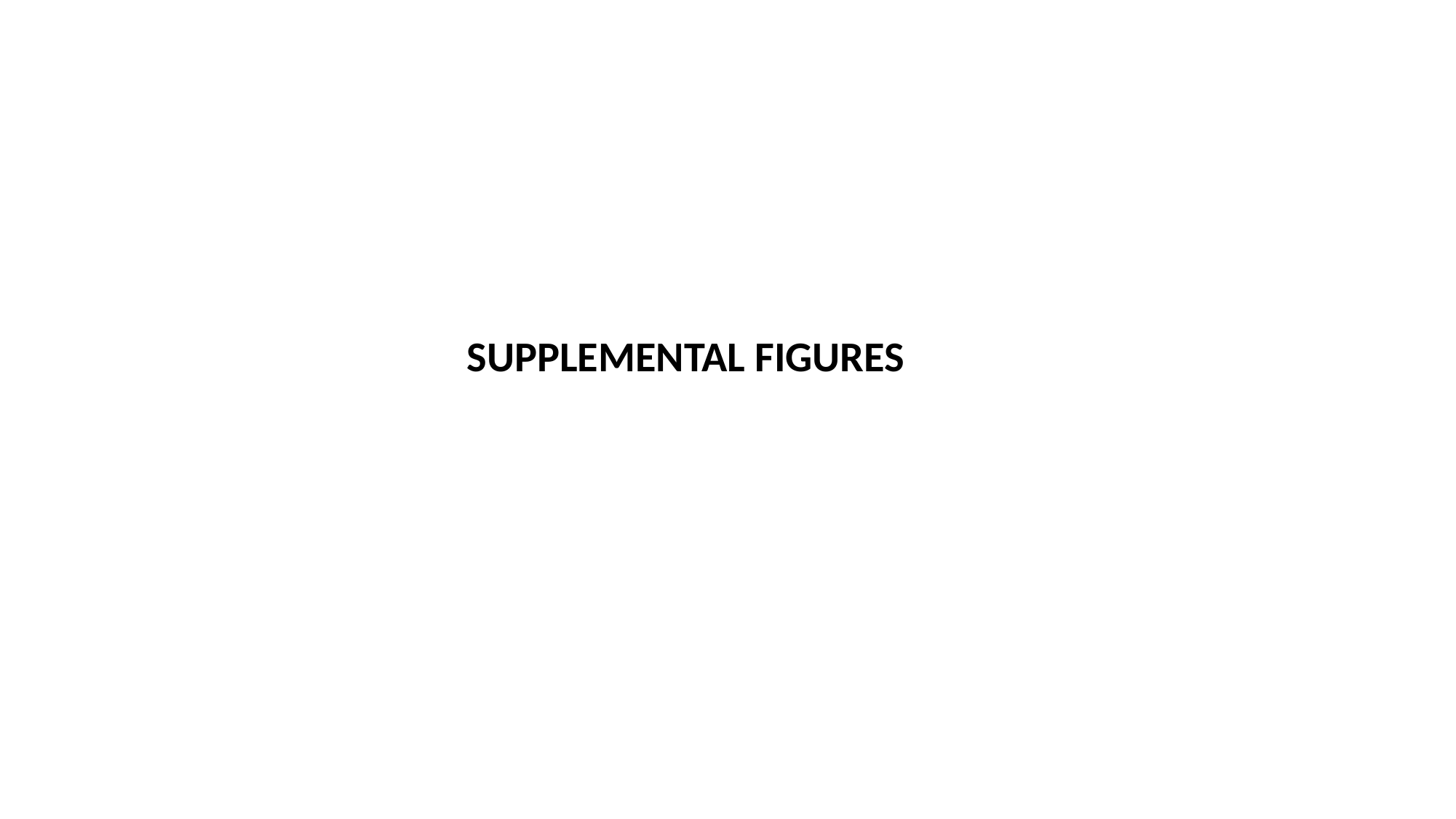

SUPPLEMENTAL FIGURES

## Slide 2
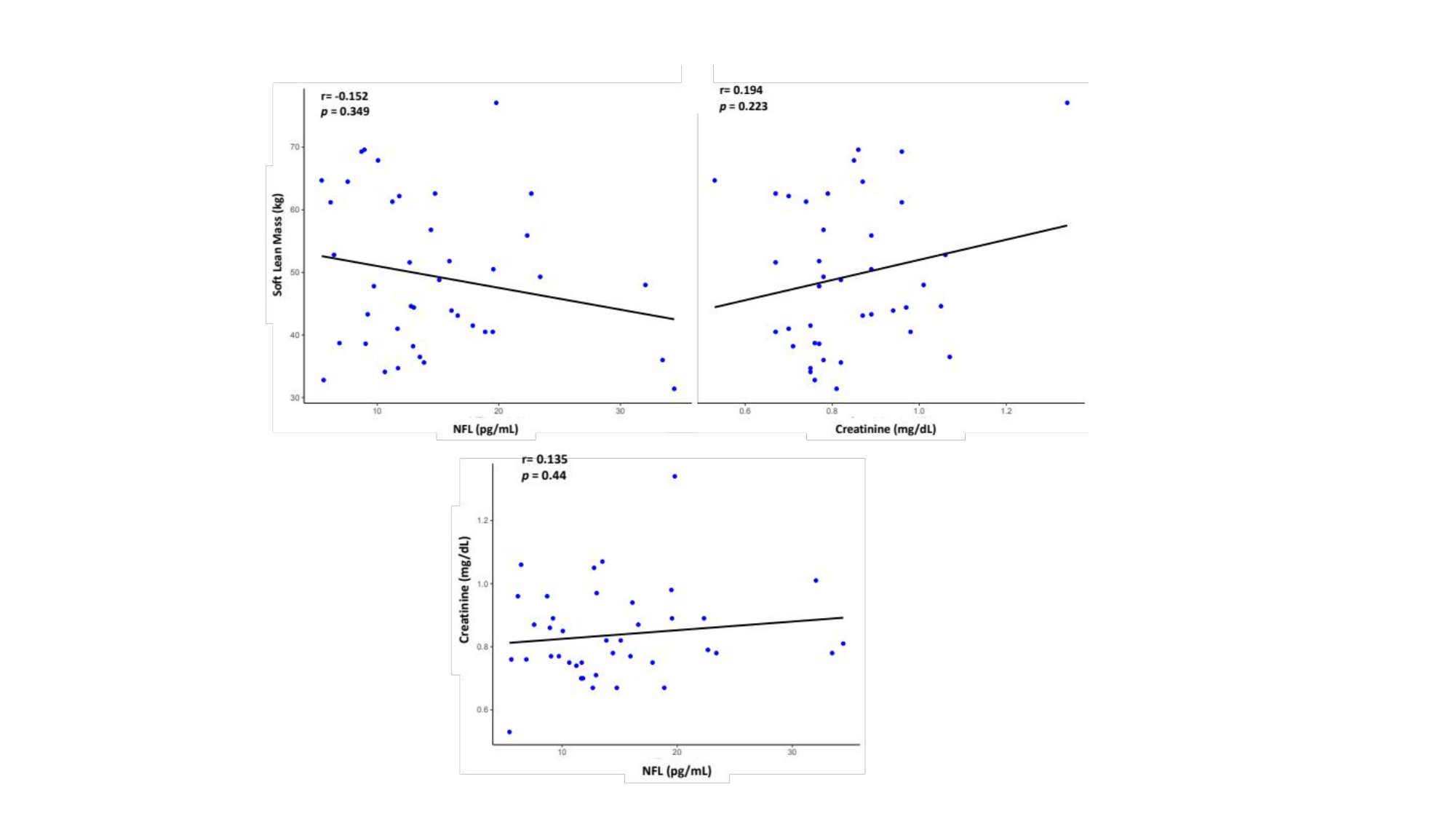

## Slide 3
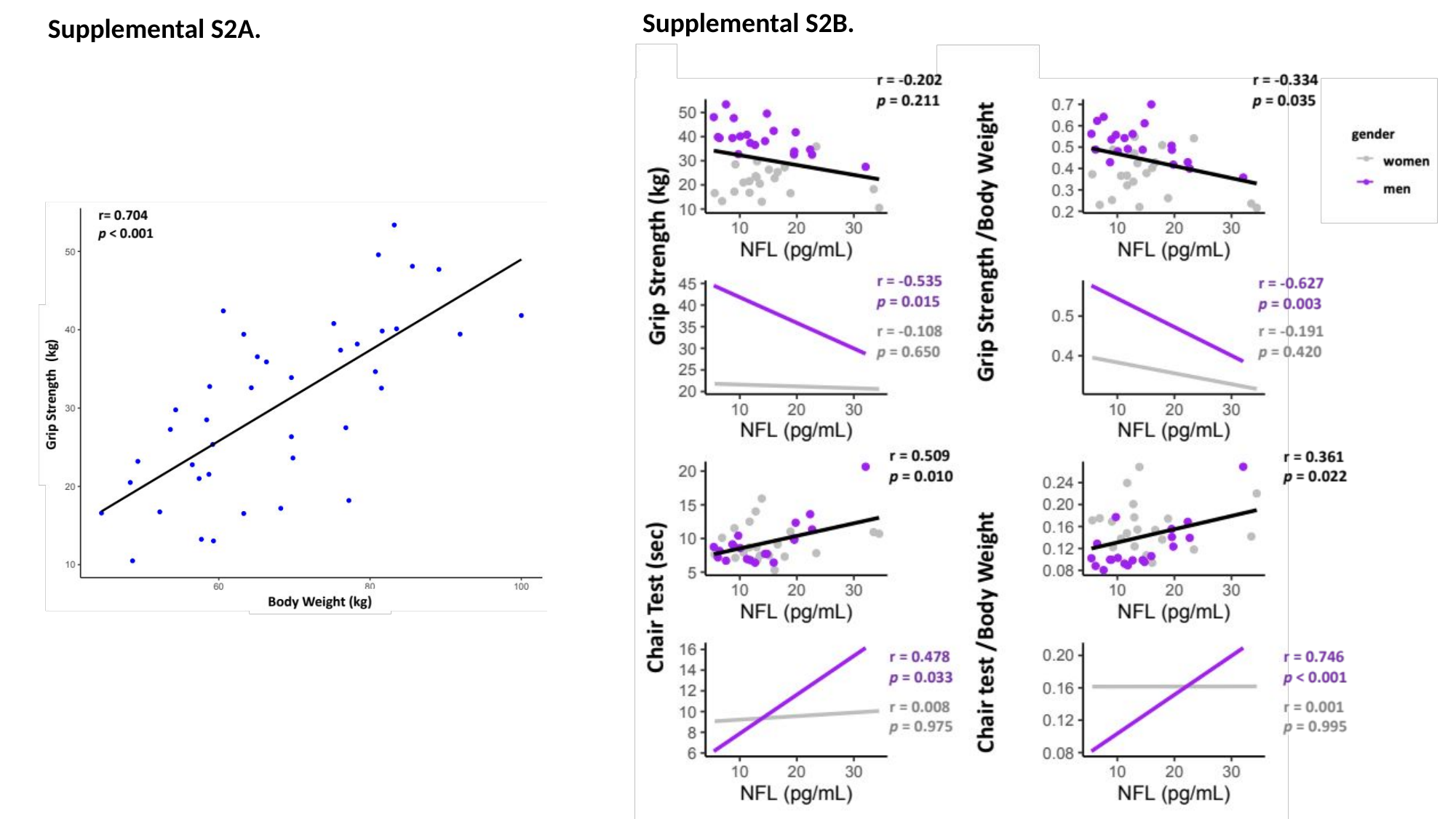

Supplemental S2B.
Supplemental S2A.

Supplement: Supplementary file 1 [file ijms-24-12751-s001.zip › Supplemental_Figures_v2.pptx]
